# Supplementary material for: Reciprocal analyses in zebrafish and medaka reveal that harnessing the immune response promotes cardiac regeneration
Source: eLife. 2017 Jun 20;6:e25605. doi: 10.7554/eLife.25605 (PMC5498136; doi:10.7554/eLife.25605)
Supplement: Supplementary file 3. — (Z-zebrafish, M-medaka, H-hours post injury, D-days post injury.) DOI: http://dx.doi.org/10.7554/eLife.25605.025 [file elife-25605-supp3.docx]

**Supplementary file 3.** **Top canonical pathways and upstream regulators predicted by Ingenuity Pathway Analysis.** (Z-zebrafish, M-medaka, H-hours post injury, D-days post injury.)

| **Canonical Pathway** | Z-6H | Z-1D | Z-2D | Z-3D | Z-5D |  | M-6H | M-2D | M-3D | M-6D |
| --- | --- | --- | --- | --- | --- | --- | --- | --- | --- | --- |
| Fcγ Receptor-mediated Phagocytosis | 3.5 | 3.8 | 3.8 | 4.2 | 4.8 |  | 0.0 | 1.1 | 1.0 | 3.5 |
| NF-κB Activation by Viruses | 1.5 | 2.4 | 2.6 | 2.4 | 2.8 |  | 2.1 | 0.0 | 1.4 | 1.5 |
| PI3K/AKT Signaling | 3.0 | 2.8 | 3.5 | 2.2 | 1.7 |  | 1.2 | -0.9 | 0.5 | 3.0 |
| Integrin Signaling | 3.3 | 2.2 | 1.1 | 3.9 | 4.9 |  | 2.1 | -0.3 | 1.9 | 1.5 |
| NFAT in Immune Response | 1.1 | 2.0 | 3.2 | 3.0 | 4.2 |  | 1.9 | 2.1 | 2.2 | 1.1 |
| *Toll-like Receptor Signaling* | 2.1 | 0.8 | 1.7 | 1.7 | 3.2 |  | 0.7 |  | 1.9 | 2.1 |
| Neuregulin Signaling | 1.3 | 2.3 | 2.6 | 2.5 | 3.3 |  | 0.0 | 0.4 | 0.5 | 1.3 |
| Telomerase Signaling | 0.7 | 2.1 | 1.7 | 2.3 | 2.3 |  | 0.0 | -0.8 | 0.3 | 0.7 |
| Wnt/β-catenin Signaling | -0.7 | 1.6 | -0.6 | 0.4 | 1.2 |  | 0.0 | -1.6 | -2.2 | -0.7 |
| HIPPO signaling | -3.0 | -1.2 | -2.1 | 0.0 | 0.0 |  |  | 0.0 | 0.7 | -3.1 |
|  |  |  |  |  |  |  |  |  |  |  |
| **Upstream regulators** | Z-6H | Z-1D | Z-2D | Z-3D | Z-5D |  | M-6H | M-2D | M-3D | M-6D |
| Poly rI:rC-RNA (TLR3 agonist) | 2.6 | 3.9 | 2.9 | 4.6 | 5.3 |  | 1.5 |  | 1.8 | 1.8 |
| CpG ODN 1826 (TLR9 agonist) | 1.9 | 2.1 | 2.4 | 2.1 | 3.1 |  |  |  |  |  |
| IFNG | 3.7 | 5.4 | 4.4 | 6.1 | 5.8 |  | 4.5 | 3.0 | 3.7 | 2.3 |
| RET | 2.7 | 2.2 | 2.5 | 3.3 | 2.2 |  | -0.4 | -0.2 | 0.2 | -0.2 |
| TGFB1 | 6.1 | 3.4 | 4.7 | 7.0 | 7.1 |  | 2.1 | 3.8 | 4.5 | 4.8 |
| IGF1 | 4.3 | 2.8 | 1.8 | 3.8 | 5.0 |  | 1.5 | 2.1 | 2.0 | 1.8 |
| MITF | -1.9 | 3.8 | 4.7 | 4.7 | 5.5 |  | 0.3 | -0.9 | 3.0 | 3.5 |
| KLF4 |  | -1.3 | -0.8 | -1.1 | -0.7 |  | 2.4 | 2.0 | 1.7 | 2.3 |
| miR-125b-5p | -2.9 | -1.8 | -4.1 | -1.4 | -2.9 |  |  |  | -0.2 |  |
| miR101 | -2.4 | -1.2 | -2.4 | -2.0 | -2.2 |  |  |  |  |  |
